# Surface Plasmon Resonance-Based Gold-Coated Hollow-Core Negative Curvature Optical Fiber Sensor

**DOI:** 10.3390/bios13020148

**Published:** 2023-01-17

**Authors:** J. Divya, S. Selvendran

**Affiliations:** School of Electronics Engineering (SENSE), Vellore Institute of Technology, Chennai 600127, Tamil Nadu, India

**Keywords:** photonic crystal fibers, hollow core, negative curvature, surface plasmon resonance, gold, sensitivity

## Abstract

The hollow-core fiber-based sensor has garnered high interest due to its simple structure and low transmission loss. A new hollow-core negative-curvature fiber (HC-NCF) sensor based on the surface plasmon resonance (SPR) technique is proposed in this work. The cladding region is composed of six circular silica tubes and two elliptical silica tubes to reduce fabrication complexity. Chemically stable gold is used as a plasmonic material on the inner wall of the sensor structure to induce the SPR effect. The proposed sensor detects a minor variation in the refractive indices (RIs) of the analyte placed in the hollow core. Numerical investigations are carried out using the finite element method (FEM). Through the optimization of structural parameters, the maximum wavelength sensitivity of 6000 nm/RIU and the highest resolution of 2.5 × 10^−5^ RIU are achieved in the RI range of 1.31 to 1.36. In addition, an improved figure of merit (FOM) of 2000 RIU^−1^ for Y-polarization and 857.1 RIU^−1^ for X-polarization is obtained. Because of its simple structure, high sensitivity, high FOM, and low transmission loss, the proposed sensor can be used as a temperature sensor, a chemical sensor, and a biosensor.

## 1. Introduction

In recent years, the optical fiber has emerged as a feasible option for sensing applications because of its small size, precision, remote sensing, and high sensitivity [1]. There are numerous versions of optical fibers, including single-mode fibers [2], multimode fibers [3], and micro- and nanostructured fibers [4]. Different types of sensing approaches are used in an optical fiber, including Bragg gratings, long-period gratings, interferometers, surface plasmon resonance (SPR), and fluorescence [5]. Yang et al., developed a long-period fiber grating (LPFG) sensor for the rapid detection of Staphylococcus aureus bacteria. Bacterial adhesion and sensitivity are enhanced by poly-electrolyte coatings. This sensor has obtained a sensitivity of 0.478 ± 0.005 nm/log (colony-forming unit/mL) at a short detection time of 30 min [6]. Ivanov et al., proposed a thin-core optical fiber sensor coated with polymer monolayers. When the layer thickness increased, the resonance frequency shifted, and this structure has been used as a chemical sensor to measure the pH level [7].

The SPR-based optical fiber sensing approach is more feasible than other conventional prism-based sensing methods due to their small size, real-time sensing, high sensitivity, and high accuracy [8,9]. A surface plasmon is produced when guided light of a specific wavelength interacts with a metal surface. As a result, the core develops a leaky mode that transfers the partial energy from core mode to plasmon mode. That particular wavelength is called the resonance wavelength, and this state is known as the phase-matching condition [10]. This SPR technique detects the minute RI variation near the metal surface. Different types of metallic coatings are used in SPR-based sensors. In optical-fiber-based sensors, noble metals are coated on the lateral surface of the fiber to realize the SPR effect [11], while in nanostructured fibers, metallic arrays are developed in the fiber tip, which enhances the sensor performance by improving the light–matter interaction [12].

The performance of the sensor is strongly influenced by the SPR effect when plasmonic materials are used. In general, the SPR effect is induced by coating or filling the sensor structure with noble metals. Gold [13] and silver [14] are the two common noble metals that are most frequently used in SPR sensors because of their sharp resonance peak, low material loss, and excellent sensitivity. Many researchers prefer gold over silver due to its higher chemical stability and biocompatibility. The two basic types of sensing techniques that can be used to find RI variations are internal and external sensing techniques. In the external sensing method, the analyte is placed on the outer surface of the sensor structure, which leads high transmission loss. To overcome this issue, the internal sensing method is used, in which the analyte is infiltered inside a hollow-core fiber, and this method reduces transmission loss and improves sensitivity by having light pass through the hollow core.

The photonic crystal fiber (PCF) is another form of optical fiber. Unlike conventional fibers, the PCF has unique properties, such as a flexible structure, strong nonlinearity, high birefringence, low transmission loss, and control sensing [15]. The limitations of conventional fiber sensors are overcome by combining PCFs with SPR techniques. Compactness and performance tuning using structural parameters of the PCF are the key benefits of SPR-based PCF sensors [16]. Depending on the guiding mechanism, the PCF has been categorized into two major types: solid-core PCF and hollow-core PCF. In solid-core fibers, light is guided by a total internal reflection method and the hollow-core fiber (HCF) drives the light inside the air core using the photonic bandgap method [17].

In an HCF, lights are guided inside the hollow core, which reduces transmission loss, which provides better accuracy and a wide transmission band [18]. In an HCF, the central air core is encased by air tubes of varying wall thicknesses. Momota et al. designed a circular-lattice hollow-core SPR-based refractive index sensor in which silver was used as a plasmonic material to induce the SPR effect, and it was placed on the outer surface of the structure. The highest wavelength sensitivity of 4200 nm/RIU was obtained for analyte RI value ranges between 1.33 and 1.37 [19]. Nazeri et al., developed a hollow-core photonic crystal fiber sensor for gas detection. This sensor obtained a maximum wavelength sensitivity of 4629 nm/RIU for the RI range of 1.0000347–1.000436 [20].

Hollow-core negative-curvature fibers (HC-NCFs) are another form of HCFs with a simple cladding region consisting of a single layer of circular or elliptical air tubes [21]. In HC-NCFs, the surface normal to the boundary is in the opposite direction from the core, which minimizes the coupling between core and cladding, which results in minimal transmission loss [22]. This type of fiber is used in remote sensing, chemical applications [23], and biosensing applications [24]. The HC-NCF has many advantages over the HCF, which include low transmission loss, a simple structure, and a large bandwidth.

Qiu et al., proposed a hollow-core negative-curvature fiber based on the surface plasmon resonance method for refractive index sensing. The cladding region was composed of eight circular silica tubes, two of which were filled with gold to produce the SPR effect. The optimum wavelength sensitivity of 5700 nm/RIU was achieved for analytes with RI values between 1.2 and 1.34 [25].

In this paper, a new HC-NCF sensor based on the SPR technique is proposed. A minimal number of silica tubes, such as six circular and two elliptical silica tubes, were preferred in the cladding region, which minimized fabrication complexity. Gold was used as a plasmonic material to realize the SPR effect. The proposed sensor is capable of detecting the RI variation in the substance present in the hollow-core region. Numerical analyses were carried out using the finite element method (FEM) in the frequency domain, and the confinement loss obtained for the proposed SPR sensor was 279.69 dB/cm and 376.83 dB/cm for X-polarization and Y-polarization, respectively. For the RI range of 1.31 to 1.36, a measured wavelength sensitivity of 6000 nm/RIU and a resolution of 2.5 × 10^−5^ RIU were obtained by the optimization of structural parameters. The proposed sensor can be used as a temperature sensor, a chemical sensor, a biosensor, and a gas sensor due to its simple structure, high sensitivity, high FOM, and low transmission loss.

## 2. Materials and Methods

**Figure 1:**
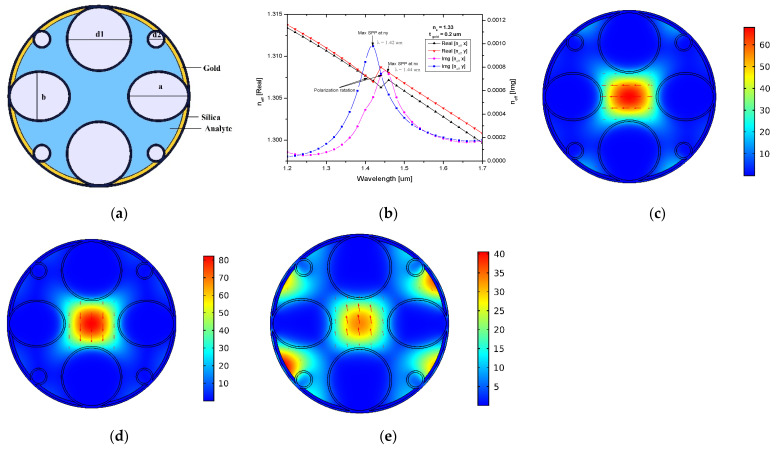
(**a**) Graphical representation of the proposed HC-NCF sensor. (**b**) Dispersion relations of the core-guided mode as a function of wavelength. Electric field distribution of (**c**) X-polarized mode, (**d**) Y-polarized mode, and (**e**) plasmon mode.

The graphical representation of the proposed SPR-based HC-NCF sensor structure is illustrated in Figure 1a. The cladding region was composed of six circular silica tubes and two elliptical silica tubes to reduce fabrication complexity. A higher birefringence was generated by using elliptical tubes; their sensing capabilities outperform those of circular ones [26]. This birefringence concept facilitates the fiber to function as a polarization filter. The analyte was filled in that structure, and it was used as a sensor to realize the resonance shift of X- and Y-polarization in different wavelengths. To generate birefringence, circular silica tubes with two different diameters, such as d1 of 3.4 µm and d2 of 1 µm, and an elliptical silica tube with major (a) and minor (b) axis diameters of 3.4 µm and 2.8 µm were used. The significant birefringence of the core separates the X- and Y-polarized modes [27]. For all circular and elliptical silica tubes, the same 0.1 µm thickness (t_silica_) was maintained. A 0.2-µm-thick (t_gold_) gold layer was coated on the inner wall of the structure to induce the SPR effect, because gold is chemically balanced and biocompatible. The outer diameter of the sensor structure was 9.8 µm, with a 0.1 µm thickness. The length of the fiber sensor was taken as 1 mm, because in photonics, the coupling length between two modes is below 6 mm [28]. The fabrication of HC-NCFs is similar to that of conventional PCFs. The two-stage stack-and-draw technique [29] was used to fabricate the proposed sensor. In the first stage, capillaries and spacing elements were stacked into a small jacket tube to create a preform, which was then pulled into canes. The second stage preform was made by inserting the cane into a large tube with an outer diameter of 9.8 µm. Different gas pressures were used in the preform to obtain a different size of hollow tubes. After assembling the fiber framework, a 0.2-µm-thick layer of gold was deposited on the inner wall of the fiber using the electroless plating technique [30] or the high-pressure chemical vapor deposition method [31]. The analyte was injected into the sensor using an infiltration method [32].

Numerical analyses were carried out using the finite element method (FEM) in the frequency domain. The amount of energy reflected was further decreased by applying a scattering boundary condition to the outer surface of the structure.

The dispersion relationship between the real and the imaginary part of the effective mode index (neff) with respect to the wavelength is shown in Figure 1b. The effective index of the real part decreased when the wavelength increased. A sharp drop in the real part of X- and Y-polarization indicates that light in the core mode is coupled to the plasmon mode. The resonance peak is the point at which the effective index of imaginary value reaches its highest value. Y-polarization reached its resonance peak at a wavelength of 1.42 µm, whereas X-polarization was minimal at this time, which shows that only Y-polarization propagated at this time, while X-polarization diminished. X-polarization reached its maximum at 1.44 µm; however, Y-polarization was attenuated in this situation. Figure 1c–e represents the electric field distribution of the X-polarized mode, the Y-polarized mode, and the plasmon mode, where incident light was guided via the core. The maximum amount of energy was transferred from core mode to plasmon mode when the phase-matching condition was achieved.

The proposed HC-NCF sensor was made up of pure silica, and the RI of pure silica was obtained using the Sellmeier equation [10]:

(1) $$n{\left(\lambda \right)}^{2}=1+\frac{A_{1}\lambda^{2}}{\lambda^{2}-\lambda_{1}^{2}}+\frac{A_{2}\lambda^{2}}{\lambda^{2}-\lambda_{2}^{2}}+\frac{A_{3}\lambda^{2}}{\lambda^{2}-\lambda_{3}^{2}}$$

where A_1_ = 0.6961663, A_2_ = 0.4079426, A_3_ = 0.897479, *λ*_1_ = 0.068404, *λ*_2_ = 0.1162414, and *λ*_3_ = 9.896161 are the Sellmeier constants and *λ* is the operating wavelength.

The Drude model was used to estimate the wavelength-dependent dispersion phenomenon of gold [10] and is expressed by

(2) $$\varepsilon =1-\frac{\omega_{P}^{\;\;\;2}}{\omega^{2}+i\omega \Gamma_{P}}$$

where the plasma frequency $\omega_{P}$ = 9.06 eV and the damping rate $\Gamma_{P}$ = 0.07 eV.

The confinement loss refers the amount of light that leaks when light is guided in the core region [33]. The following equation is used to calculate the confinement loss:

(3) Confinement loss=8.686×2Πλ×Im(neff)×104(dB/cm),

where Im (n_eff_) is the imaginary part of the effective mode index and *λ* is the operating wavelength.

## 3. Results

The performance if the proposed SPR-based HC-NCF sensor was evaluated by optimizing geometrical parameters, such as the gold layer thickness and the diameter of the circular and elliptical silica tubes. The sensor’s performance was enhanced when these parameters were optimized. The impact of each parameter was investigated by individually varying it, while maintaining the other structural elements constant, and a constant RI value of 1.33 was used for the analyte throughout the optimization procedure.

**Figure 2:**
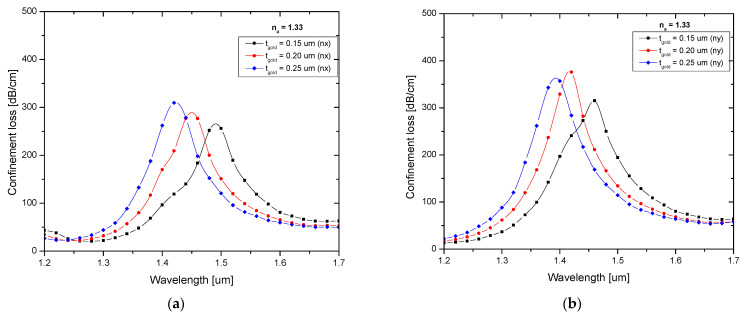
Confinement loss characteristics for different gold layer thicknesses for (**a**) X-polarization and (**b**) Y-polarization.

The gold layer thickness, as well as the resonance wavelength, had a substantial impact on the sensor’s performance. The effects of various gold layer thicknesses, including 0.15 µm, 0.2 µm, and 0.25 µm, were analyzed, Figure 2a,b shows the confinement loss spectrum for X-polarization and Y-polarization. The resonance peak shifted to shorter wavelengths when the gold layer thickness increased. The best sensitivity was obtained by using the strongest resonance peak, which could be seen at a thickness of 0.2 µm. The effectiveness of a sensor is dependent on its sensitivity. The following expression can be used to calculate the sensor’s wavelength sensitivity [10]:

(4) $$s_{\lambda \;}=\frac{\Delta \lambda_{Peak}}{\Delta n_{a}}\;(\mathrm{nm}/\mathrm{RIU})$$

**Figure 3:**
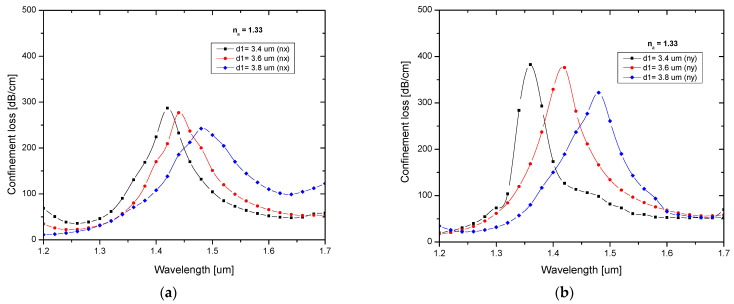
Confinement loss characteristics for different values of d1 for (**a**) X-polarization and (**b**) Y-polarization.

The circular and elliptical silica tube diameter plays a vital role in the sensor’s sensing performance because these silica tubes create the path between core and analyte. In the cladding region, elliptical as well as circular silica tubes combine to form an anisotropic shape. An anisotropic shape provides a strong negative curvature and a node-free anti-resonance element [34], and this structure also enhances sensor performance. Figure 3a,b depicts the loss spectrum for different values of d1 for X-polarization and Y-polarization. The core effective area decreased as the silica tube radius increased, which in turn decreased the core strength. This shows that as the silica tube diameter increases, the resonance peak shifts to longer wavelengths. At a diameter of 3.4 µm, a sharp peak was obtained for both polarizations and was used for further analysis.

**Figure 4:**
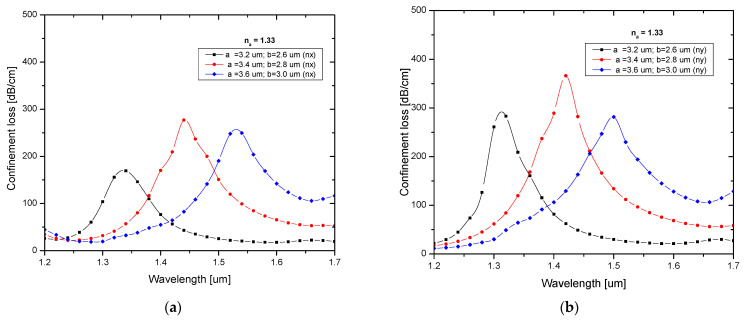
Confinement loss characteristics of different major and minor axes for (**a**) X-polarization and (**b**) Y-polarization.

The loss spectrum for various major and minor axes of the elliptical tube for X-polarization and Y-polarization is shown in Figure 4a,b. The core effective area decreased as the elliptical tube’s major axis increased, which in turn decreased the core strength. This shows that as the elliptical tube major and minor axes increase, the resonance peak shifts to longer wavelengths. A sharp loss peak was observed for both polarizations at 3.4 µm for the major and 2.8 µm for the minor axis.

**Figure 5:**
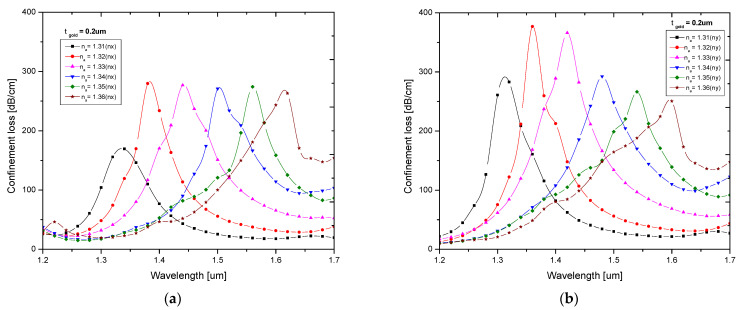
Confinement loss characteristics with respect to wavelength for RI ranges of 1.31–1.36 for (**a**) X-polarization and (**b**) Y-polarization.

To evaluate the proposed sensor, the confinement loss properties of various analytes, which had a range of 1.31 to 1.36 with a step value of 0.01, were analyzed. Figure 5a,b depicts the loss spectrum for various analyte RI values for X-polarization and Y-polarization.

As shown in Figure 5a,b, the resonance peak moved to longer wavelengths as the RI value increased. For various RI values, Y-polarization also exhibited greater confinement loss. The maximum confinement loss of 376.83 dB/cm was found for an analyte RI value of 1.32 at a resonance wavelength of 1.36 µm. In contrast to Y-polarization, X-polarization experienced less confinement loss. The maximum confinement loss of 279.69 dB/cm was observed at a resonance wavelength 1.38 µm, where the RI value was 1.32. Table 1 shows the performance analysis results of the proposed sensor for different analyte RI values for X- and Y-polarization.

The proposed sensor’s resolution was a measurement of its capacity to identify even the slightest fluctuations in the analyte RI. The following expression can be used to calculate the resolution of the sensor [10]:

(5) $$\mathrm{Resolution}=\Delta n_{a}\times \frac{\Delta \lambda_{min}}{\Delta \lambda_{Peak}}\;(\mathrm{RIU})$$

where Δ*λ_min_* represents the minimum spectral resolution, and this was taken as 0.1 nm. Δ*λ_peak_* is the peak wavelength shift, and Δn_a_ is the change of the analyte RI. The highest resolution measured using this sensor was 2.5 × 10^−5^ RIU.

The figure of merit (FOM) is another significant sensor characteristic. This is a wavelength-dependent parameter, and it is defined as the ratio between the sensitivity and the full width half maximum (FWHM), which was obtained from the following equation [10]:

(6) $$\mathrm{FOM}=\frac{S\;\left(\mathrm{nm}/\mathrm{RIU}\right)}{FWHM\;\left(\mathrm{nm}\right)}\;({\mathrm{RIU}}^{-1})$$

**Figure 6:**
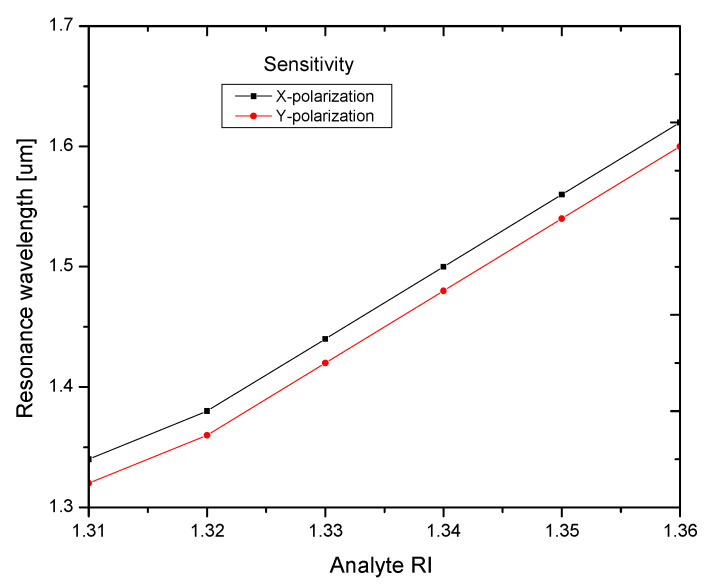
Sensitivity analysis of different analyte RI values for X- and Y-polarization.

The sensitivity of the proposed sensor for X- and Y-polarization is depicted in Figure 6. It shows that a maximal sensitivity of 6000 nm/RIU was obtained in the RI range of 1.31 to 1.36. The proposed sensor performed better than those described in the recent literature. Table 2 shows a performance comparison between the proposed sensor and the most recent publications.

Comparing the obtained result with experimentally investigated HCF-based sensors, the proposed sensor performed better than previously reported sensors in terms of sensitivity, FOM, resolution, and confinement loss [38,39].

## 4. Conclusions

A new SPR-based hollow-core negative-curvature fiber (HC-NCF) sensor was proposed. The cladding region was formed by six circular silica tubes and two elliptical silica tubes to reduce fabrication complexity. Both elliptical and circular silica tubes were combined to generate an anisotropic shape, which outperformed an isotropic sensor in terms of performance. A high birefringence was created using two different sizes of circular silica tubes. Chemically stable gold was used as a plasmonic material on the inner wall of the sensor structure to induce the SPR effect. The proposed sensor detected the minor variation in the RI of the analytes placed in the hollow core. Numerical analyses were carried out using the FEM in the frequency domain, and the confinement loss obtained for the proposed SPR sensor was 279.69 dB/cm for X-polarization and 376.83 dB/cm for Y-polarization. The highest resolution of 2.5 × 10^−5^ RIU and the maximum wavelength sensitivity of 6000 nm/RIU were accomplished in the RI range of 1.31 to 1.36 through the optimization of structural parameters. In addition, a FOM value of 2000 RIU^−1^ for Y-polarization and 857.1 RIU^−1^ for X-polarization was realized. Due to high confinement loss and FOM, Y-polarization was used for better performance of the proposed sensor. Because of its simple structure, high sensitivity, high FOM, and low transmission loss, this sensor can be used as a temperature sensor, a chemical sensor, and a biosensor.

## Figures and Tables

**Table 1:** Sensitivity analysis for different analyte RI values.

Refractive Index	X-Polarization				Y-Polarization				Refractive Index Resolution (RIU)	Sensitivity (nm/RIU)
	Resonance Wavelength (µm)	Confinement Loss (dB/cm)	FWHM (nm)	FOM (RIU^−1^)	Resonance Wavelength (µm)	Confinement Loss (dB/cm)	FWHM (nm)	FOM (RIU^−1^)		
1.31	1.34	169.34	12	-	1.32	283.07	04	-	2.5 × 10^−5^	-
1.32	1.38	279.69	05	800	1.36	376.83	02	2000	1.67 × 10^−5^	4000
1.33	1.44	276.90	09	666.7	1.42	366.07	03	2000	1.67 × 10^−5^	6000
1.34	1.5	270.94	08	750	1.48	292.27	05	1200	1.67 × 10^−5^	6000
1.35	1.56	274.13	07	857	1.54	266.36	05	1200	1.67 × 10^−5^	6000
1.36	1.62	262.97	09	666.7	1.6	250.58	07	857.1	-	6000

**Table 2:** Comparative study of the sensor’s performance and sensors in other recent published work.

Ref.	Structures	Material Used	Sensitivity (nm/RIU)	RI Range
[19]	Hollow-core PCF	Silver	4200	1.33–1.37
[20]	Hollow-core PCF	-	4629	1.000034–1.000449
[25]	Hollow-core NCF	Gold	5700	1.2–1.34
[35]	Hollow-core graded-index fiber	Silver	4350	1.38–1.49
[36]	Hollow-core micro-structured fiber	-	3000	1.325–1.36
[37]	Negative-curvature HC fiber	-	4411	1.33–1.39
Proposed work	Hollow-core NCF	Gold	6000	1.31–1.36

## Data Availability

Not applicable.
